# Arterivirus nsp12 versus the coronavirus nsp16 2′-*O*-methyltransferase: comparison of the C-terminal cleavage products of two nidovirus pp1ab polyproteins

**DOI:** 10.1099/vir.0.000209

**Published:** 2015-09

**Authors:** Kathleen C. Lehmann, Lisa Hooghiemstra, Anastasia Gulyaeva, Dmitry V. Samborskiy, Jessika C. Zevenhoven-Dobbe, Eric J. Snijder, Alexander E. Gorbalenya, Clara C. Posthuma

**Affiliations:** ^1^​ Department of Medical Microbiology, Leiden University Medical Center, 2300 RC, Leiden, The Netherlands; ^2^​ Belozersky Institute of Physico-Chemical Biology, Lomonosov Moscow State University, 119899 Moscow, Russia; ^3^​ Faculty of Bioengineering and Bioinformatics, Lomonosov Moscow State University, 119899 Moscow, Russia

## Abstract

The 3′-terminal domain of the most conserved ORF1b in three of the four families of the order *Nidovirales* (except for the family *Arteriviridae*) encodes a (putative) 2′-*O*-methyltransferase (2′-*O*-MTase), known as non structural protein (nsp) 16 in the family *Coronaviridae* and implicated in methylation of the 5′ cap structure of nidoviral mRNAs. As with coronavirus transcripts, arterivirus mRNAs are assumed to possess a 5′ cap although no candidate MTases have been identified thus far. To address this knowledge gap, we analysed the uncharacterized nsp12 of arteriviruses, which occupies the ORF1b position equivalent to that of the nidovirus 2′-O-MTase (coronavirus nsp16). In our in-depth bioinformatics analysis of nsp12, the protein was confirmed to be family specific whilst having diverged much further than other nidovirus ORF1b-encoded proteins, including those of the family *Coronaviridae*. Only one invariant and several partially conserved, predominantly aromatic residues were identified in nsp12, which may adopt a structure with alternating α-helices and β-strands, an organization also found in known MTases. However, no statistically significant similarity was found between nsp12 and the twofold larger coronavirus nsp16, nor could we detect MTase activity in biochemical assays using recombinant equine arteritis virus (EAV) nsp12. Our further analysis established that this subunit is essential for replication of this prototypic arterivirus. Using reverse genetics, we assessed the impact of 25 substitutions at 14 positions, yielding virus phenotypes ranging from WT-like to non-viable. Notably, replacement of the invariant phenylalanine 109 with tyrosine was lethal. We concluded that nsp12 plays an essential role during EAV replication, possibly by acting as a co-factor for another enzyme.

## Introduction

**Fig. 1.**
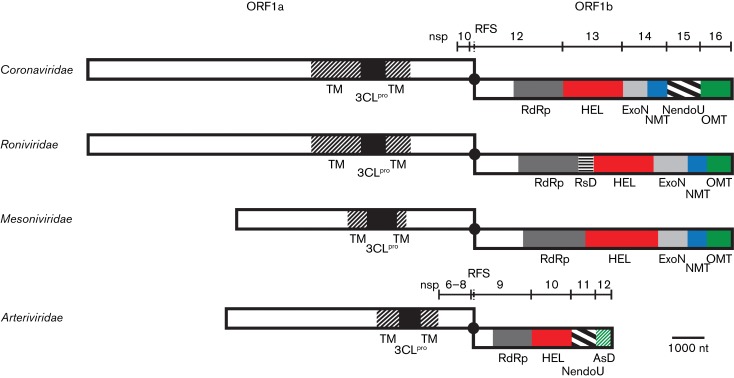
Organization of key replicase domains encoded by nidovirus ORFs 1a and 1b. Proteolytic cleavage products described in the text for the coronaviruses and arteriviruses are indicated. Matching colours/patterns indicate domain conservation between families. Domains (putatively) involved in capping (HEL, NMT, *O*MT and AsD) are depicted in bright colours. TM, transmembrane domain; 3CL^pro^, 3C-like protease; black dot and RFS, ribosomal frameshift site; RdRp, RNA-dependent RNA polymerase; HEL, ZBD, helicase core (HEL1) and variable additional domains; ExoN, exoribonuclease; NMT, N7-MTase; NendoU, endoribonuclease; OMT, 2′-*O*-MTase; RsD, ronivirus-specific domain; AsD, arterivirus-specific domain (nsp12). Genomic organizations are shown for Beluga whale coronavirus SW1 (family *Coronaviridae*), Gill-associated virus (family *Roniviridae*), Nam Dinh virus (family *Mesoniviridae*) and PRRSV, North American genotype (family *Arteriviridae*). Depicted is a simplified domain organization as most enzymes are multidomain proteins. Note that viruses of the family *Coronaviridae* that do not belong to the subfamily *Coronavirinae* encode a truncated version of NMT. Adapted from Lauber *et al.* (2013).

Members of the family *Arteriviridae* are positive-stranded RNA viruses with genome sizes ranging from 13 to 16 kb. The family currently comprises a single genus (*Arterivirus*) that includes four species: *Equine arteritis virus* (EAV), *Simian hemorrhagic fever virus* (SHFV), *Lactate dehydrogenase-elevating virus* (LDV) and *Porcine reproductive and respiratory syndrome virus* (PRRSV) (Faaberg *et al.*, 2012; Snijder *et al.*, 2013). Amongst those, the latter is the economically most relevant species causing annual losses to the American swine industry alone of ∼$800 million (Sang *et al.*, 2014). Additionally, several recently identified arteriviruses remain to be formally classified, but are likely to prototype multiple novel species or even higher-order taxa (Bailey *et al.*, 2014; Dunowska *et al.*, 2012; Lauck *et al.*, 2011, 2013). Arterivirus genomes are polycistronic and contain 10–15 (known) ORFs. The 5′-proximal ORFs 1a and 1b are expressed as polyproteins (pps) 1a and 1ab that are autoproteolytically processed into the non structural proteins (nsps) required for genome replication and transcription (Fig. 1) (Molenkamp *et al.*, 2000). The remaining ORFs mostly encode structural proteins that are expressed from a set of subgenomic mRNAs (Pasternak *et al.*, 2006). Based on overall similarities in terms of genome expression and organization, as well as synteny and homology of key replicase domains, the family *Arteriviridae* was united in the order *Nidovirales* with the families *Mesoniviridae*, *Roniviridae* and *Coronaviridae*, the latter including two distantly related subfamilies, *Coronavirinae* and *Torovirinae* (de Groot *et al.*, 2012a, b). In the nidovirus tree, the arteriviruses form a basal lineage next to the one that combines the three other families, which have substantially larger genomes (Nga *et al.*, 2011).

ORF1b is the most conserved part of the nidovirus genome and all ORF1b-encoded proteins characterized thus far are enzymes conserved in two or more nidovirus families. The RNA-dependent RNA polymerase and a zinc-binding domain (ZBD) fused with a superfamily 1 helicase (HEL1) are conserved in all nidoviruses. In contrast, six other domains are lineage specific. Four of these are conserved in two or three nidovirus families only: exoribonuclease (ExoN), N7-methyltransferase (N7-MTase), nidovirus uridylate-specific endoribonuclease (NendoU) and 2′-*O*-methyltransferase (2′-*O*-MTase). Two other domains are as yet uncharacterized and unique to either roniviruses [ronivirus-specific domain (RsD)] or arteriviruses [arterivirus-specific domain (AsD)]. As five of the six lineage-specific domains occupy a unique position in the genome, the pattern of their conservation could be explained by loss or acquisition of a single domain during nidovirus evolution (Nga *et al.*, 2011). The exception is AsD, which resides in the most C-terminal subunit of the arterivirus ORF1b polyprotein (nsp12), the position occupied by the 2′-*O*-MTase protein in all other nidoviruses (nsp16 in coronaviruses; Fig. 1). If these positionally equivalent proteins are unrelated, as reported 14 years ago based on the analysis of only a few genome sequences and prior to the identification of the 2′-*O*-MTase (Gorbalenya, 2001), their emergence would require the consideration of complex evolutionary hypotheses. Thus, the relation of AsD with the 2′-*O*-MTase and other proteins must be re-evaluated whilst taking advantage of the increased availability of sequences and improved techniques.

Unlike AsD, the coronavirus 2′-*O*-MTase has been characterized experimentally (Bouvet *et al.*, 2010; Chen *et al.*, 2011; Decroly *et al.*, 2008, 2011) and was found to provide one of the four activities required for the formation of a so-called type I cap (cap-1) (mGpppNm) structure at the 5′ end of coronaviral mRNAs (Lai & Stohlman, 1981; van Vliet *et al.*, 2002). Two other coronavirus enzymes, HEL1 (nsp13) (Ivanov *et al.*, 2004; Ivanov & Ziebuhr, 2004) and the N7-MTase (nsp14) (Bouvet *et al.*, 2010; Chen *et al.*, 2009), are also suspected or known to be involved in capping, whereas the fourth enzyme required (guanylyltransferase) remains to be identified. *In vitro*, the coronavirus N7-MTase and 2′-*O*-MTase were found to cooperate during cap formation. The latter enzyme also requires the ORF1a-encoded nsp10 as a co-factor (Bouvet *et al.*, 2010). Although arteriviruses were not characterized in detail, the SHFV genome was reported to be capped (Sagripanti *et al.*, 1986) and they do encode a HEL1 (Seybert *et al.*, 2000), which could contribute to capping. Thus, the discovery of arteriviral N7-MTase and/or 2′-*O*-MTase activities could be readily accommodated in a functionally sensible manner.

Based on the above evolutionary and functional considerations, we sought to characterize nsp12 of arteriviruses by testing the hypothesis that it may be an MTase. We show that, unlike the coronavirus 2′-*O*-MTase, nsp12 is poorly conserved amongst known arteriviruses compared with the proteins carrying the endoribonuclease (nsp11) and helicase (nsp10) activities, and that it contains only one evolutionary invariant residue. No statistically significant similarity was found between arterivirus nsp12 and coronavirus nsp16 or other proteins although the two nidovirus proteins may belong to the same α/β-fold class. Likewise, no MTase activity was detected in carefully controlled assays using recombinant EAV nsp12 in the absence or presence of several other nsps that were included as potential co-factors. Using reverse genetics, a large set of EAV nsp12 mutants was generated and tested for replication, revealing phenotypes ranging from WT-like to replication-deficient, which broadly correlated with the natural variation of the probed residues. We conclude that nsp12 plays an essential role in EAV replication and discuss possible directions to elucidate its enigmatic function.

## Results

### Sequence similarities and dissimilarities between arterivirus nsp12 and (putative) MTases of the *Coronaviridae* family

**Fig. 2.**
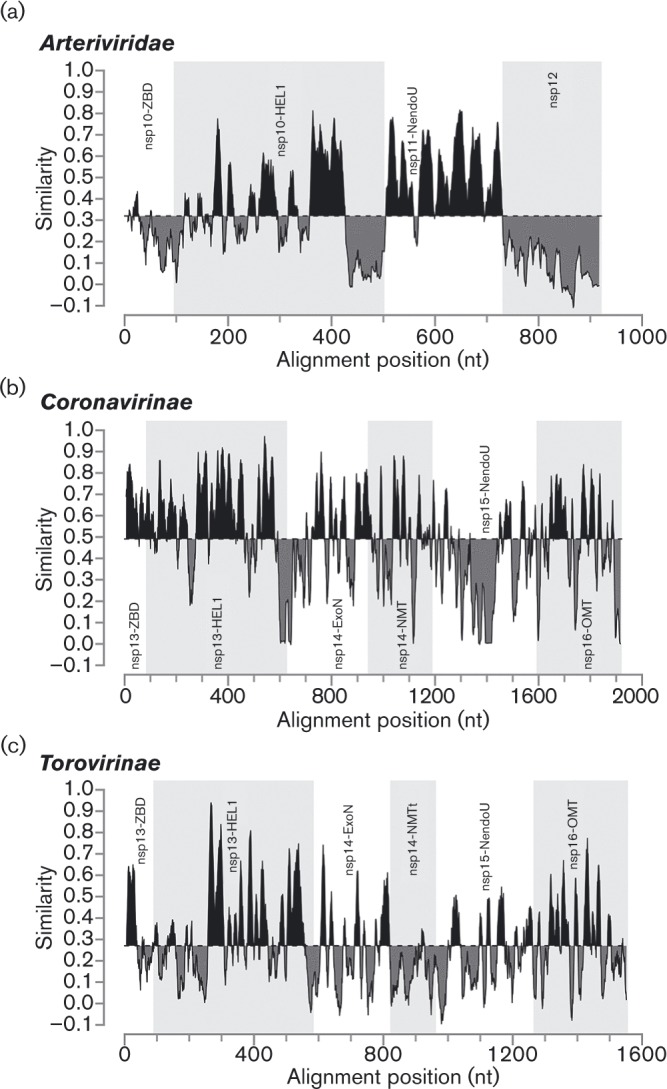
Similarity density plots of the C-terminal region of pp1ab of different nidovirus (sub)families. Values above and below average similarities are indicated in black and grey, respectively. HEL1, helicase core domain; ExoN, exoribonuclease; NMT, *N* ^7^-MTase (t, truncated); NendoU, endoribonuclease; OMT, 2′-*O*-MTase. For the sake of simplicity, we have applied the nsp nomenclature of the subfamily *Coronavirinae* also to the orthologous torovirus domains for which the processing of pp1a/pp1ab is yet to be fully described.

**Fig. 3.**
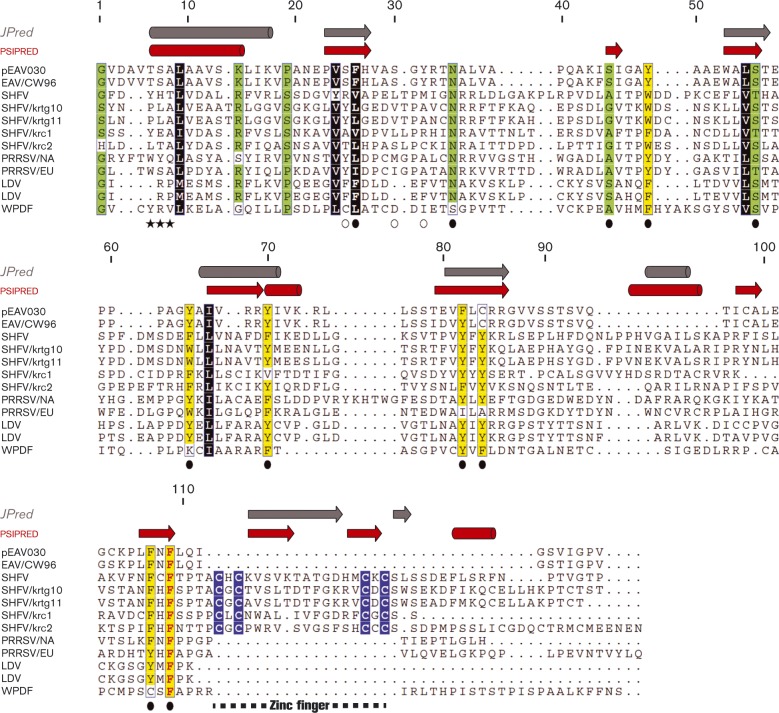
Multiple sequence alignment (MSA) and secondary structure predictions of representative arterivirus nsp12 sequences. Partially and fully conserved amino acids are highlighted in coloured boxes. Colours represent residues with similar biophysical properties; yellow, aromatic; black, hydrophobic; blue, (putative) zinc binding; green, other. Secondary structures (barrel, α-helix; arrow, β-strand) were predicted with JPred (Cole *et al.*, 2008) (grey) or psipred (Buchan *et al.*, 2013) (red) based on the MSA. Residue numbers are indicated for nsp12 of the EAV-Bucyrus isolate (pEAV030) (van Dinten *et al.*, 1997), the parental strain of pEAV211 used for the reverse genetics experiments. Replaced residues are indicated below the alignment; black stars, positions where stop codons were introduced; empty circles, control residues; filled circles, conserved residues. A putative zinc finger in simian arterivirus nsp12 sequences is indicated by a dashed line. EAV, GenBank accession number AY349167; SHFV, GenBank accession numbers AF180391, JX473847, JX473848, HQ845737 and HQ845738; PRRSV, GenBank accession numbers JX138233 and JF802085; LDV, GenBank accession numbers L13298 and U15146; WPDV, GenBank accession number JN116253.

We first analysed the conservation of nsp12 in comparison with that of other proteins deriving from the C-terminal portion of pp1ab of arteriviruses, coronaviruses and toroviruses. Starting at the ZBD, the region analysed included the three proteins implicated in 5′ cap formation in coronaviruses. We found that nsp12 was conserved in all established and provisional arterivirus species, including the most distantly related wobbly possum disease virus (WPDV). Inspection of the arterivirus conservation profile showed that the entire nsp12 sequence exhibits similarity values that are below average for this pp1ab region (0.320 on a scale from − 0.1 to 1.0; Fig. 2a). Only the C-terminal domain of nsp10 and to some extent the ZBD were similarly divergent, whilst the similarity of the nsp10 helicase core and particularly nsp11 were above average. This remarkably low conservation distinguishes arterivirus nsp12 also from all proteins in this region of the coronaviruses (average conservation 0.491) and toroviruses (0.270), including nsp16 (Fig. 2b, c). Accordingly, arterivirus nsp12 contains the smallest number of conserved residues amongst the analysed proteins, with only a single phenylalanine (F109 in EAV) being evolutionarily invariant (Fig. 3). Other notable conserved nsp12 residues (out of 18 in total) were an asparagine, a serine/threonine and six aromatic residues. We also noted the presence of four conserved cysteines in a pattern typical for zinc fingers in the C-terminal part of nsp12 in the five simian arteriviruses, which constitute a phylogenetically compact cluster. Patristic pair-wise distances (PPDs) of nsp12 compared with those of ZBD, HEL1 and NendoU were consistently larger, whilst PPDs of (putative) 2′-*O*-MTases were comparable on average with those of five other domains in the coronaviruses and toroviruses (Fig. S1, available in the online Supplementary Material). These results showed that, in comparison with the coronavirus 2′-*O*-MTase, nsp12 must have evolved under unusually relaxed constraints or in a changing molecular environment. Secondary structure predictions using JPred and psipred consistently indicated the alternation of α-helices and β-strands in arterivirus nsp12 (Fig. 3). Interestingly, the coronavirus MTases also belong to the α/β structural class and contain conserved aromatic residues (Chen *et al.*, 2011; Decroly *et al.*, 2011). Nevertheless, HH-suite profile–profile comparison did not reveal sequence similarity above the background between nsp12 and the 2′-*O*-MTase of coronaviruses or toroviruses, *E* = 0.41 and 0.53, respectively (Fig. S2). Furthermore, these proteins are also of different sizes: 119–178 (arterivirus nsp12) versus 263–312 aa (coronavirus nsp16), with the arterivirus proteins being also smaller than MTases of other origins. The above HH-based negative result contrasted with the strong similarity signal observed in (control) comparisons between arteriviruses and coronaviruses or toroviruses for HEL1 and NendoU (*E* ≤ 3.5 × 10^–17^) or in the control comparison between coronavirus and torovirus nsp16, *E* = 2.3 × 10^–32^ (Fig. S2). No statistically significant similarity was observed between nsp12 and other proteins in a hidden Markov model (HMM)-based scan of the Pfam-A database (top hit: PF12581, *E* = 1.0). We thus concluded that nsp12 has diverged beyond recognition from its homologues and differs considerably from the 2′-*O*-MTase of large nidoviruses. Nevertheless, the results obtained did not rule out the possibility that it could be a deviant MTase, and we therefore set out to test this hypothesis experimentally by biochemical and molecular virological methods.

### Purification of recombinant EAV nsp12 and several ORF1a-encoded proteins

**Fig. 4.**
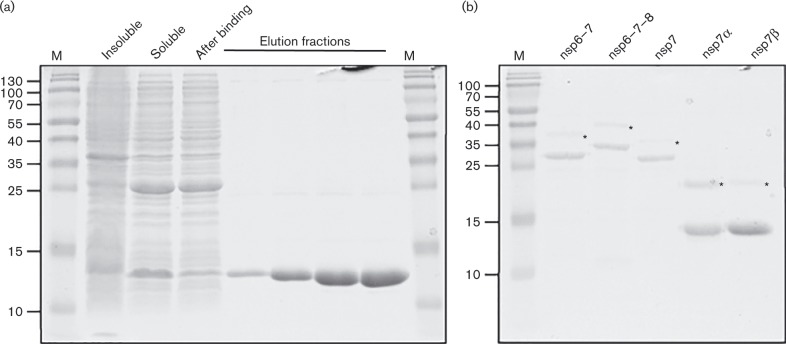
SDS-PAGE analysis of purified EAV nsps. (a) The progression of metal-ion chromatography of EAV nsp12-containing (13 kDa) *E. coli* lysates was monitored by Coomassie brilliant blue staining. Insoluble and soluble: proteins retained in pellet or supernatant, respectively, after cell lysis and ultracentrifugation; after binding: proteins in supernatant after removal of Talon beads. (b) Elution fractions of EAV ORF1a proteins and intermediates (nsp6–7, 29 kDa; nsp6–7–8, 34 kDa; nsp7, 26 kDa; nsp7α, 15 kDa; nsp7β, 13 kDa). Products marked with an asterisk are the remaining ubiquitin–nsp fusion proteins. Lane M, size markers (kDa).

We engineered vectors encoding recombinant EAV nsp12 derivatives carrying either an N-terminal or a C-terminal His6-tag and expressed them in *Escherichia coli*. Only the N-terminally tagged protein was successfully expressed and purified by metal affinity chromatography using Co^2+^ (Talon) beads (Fig. 4a). The protein appeared to be reasonably stable under all conditions tested, including a pH range from 6.0 to 7.5 and protein concentrations of up to 500 μM. Yet upon storage the protein increasingly formed dimers and higher-order multimers, even in the presence of 1 mM DTT. In gel filtration experiments with fresh protein these oligomers were not evident. Instead, a single peak was observed (not shown) that corresponded well to the expected size of an nsp12 monomer (calculated weight 13 kDa versus predicted weight based on Stokes radius 16 kDa).

In addition to nsp12, we also expressed five small mature proteins and cleavage intermediates from the nsp7 region of pp1a [nsp6–7, nsp6–7–8, nsp7α, nsp7β and nsp7 (i.e. nsp7α-7β)] (van Aken *et al.*, 2006; Wassenaar *et al.*, 1997) (Fig. 4b). In coronaviruses, the corresponding part of ORF1a encodes nsp10, an essential co-factor for the 2′-*O*-MTase (Bouvet *et al.*, 2010). Consequently, we added these purified recombinant proteins to nsp12 in MTase activity assays (see below).

### Recombinant nsp12 does not display *in vitro* MTase activity using a variety of substrates

**Fig. 5.**
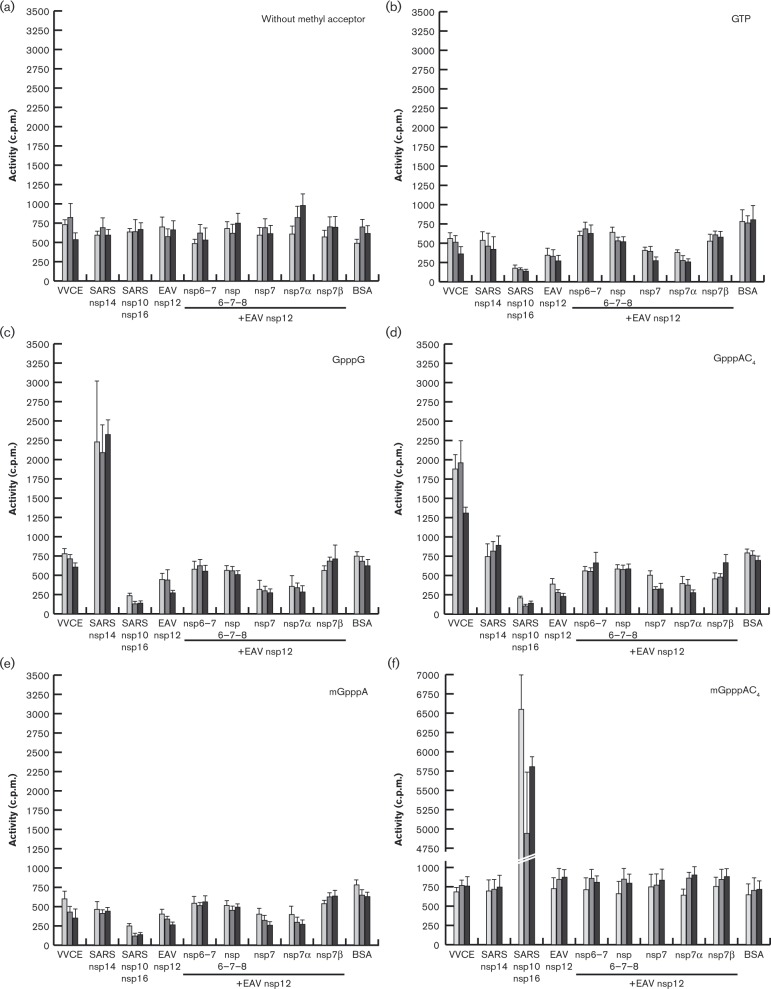
MTase activity assays using recombinant EAV nsp12 in the presence and absence of possible co-factors. Recombinant EAV nsp12 (1 μM) and equimolar amounts of the indicated possible co-factors were incubated for 30 (light grey), 60 (dark grey) or 180 min (black) with *S*-[methyl-^3^H]adenosylmethionine and the indicated methyl acceptor. Proteins with known MTase activity served as positive controls. VVCE, Vaccinia virus capping enzyme (0.1 U μl^− 1^, *N*7-MTase); SARS nsp14 (75 nM, *N* ^7^-MTase); SARS nsp10/nsp16 (2 μM complex, 2′-*O*-MTase); SARS, SARS coronavirus; BSA served as negative control. Reslults are presented as mean ± sd of two independent experiments. The background variation evident for several of the protein combinations using GTP, GpppG, GpppAC_4_ or mGpppA most likely represents an artefact originating from a position effect, which was observed repeatedly in the employed 96-well format.

Using purified arterivirus proteins, we proceeded to test for MTase activity in the presence of different methyl acceptors by employing an *in vitro* assay similar to that previously established for severe acute respiratory syndrome coronavirus (SARS-CoV) nsp14 and nsp16 (Bouvet *et al.*, 2010). In agreement with the published results (Bouvet *et al.*, 2010; Chen *et al.*, 2009; Jin *et al.*, 2013), both SARS-CoV MTases (kindly provided by Dr Etienne Decroly, AFMB, Marseille, France), which were used as positive controls, transferred the radioactive methyl group from the universal methyl donor *S*-adenosylmethionine to non-methylated or N7-methylated cap analogues (Fig. 5). Likewise, vaccinia virus capping enzyme, obtained from a commercial source and known to harbour N7-MTase activity, also demonstrated the expected activity. Based on these activities and the results of two negative control reactions (assays using BSA and no acceptor), we defined an incorporation threshold of 1000 c.p.m. to distinguish the enzyme activity in this assay. According to this definition, EAV nsp12 did not display activity with any of the methyl acceptors in the absence or presence of any of the potential ORF1a-encoded co-factors described above (nsp6–7, nsp6–7–8, nsp7α, nsp7β and nsp7). However, we can not exclude the possibility that the relatively high background may have obscured any low-level activity, if present.

### Tolerance of EAV replication to nsp12 mutagenesis correlates with the natural variation of probed residues

To establish the general importance of nsp12 for EAV replication, we used reverse genetics to assess whether EAV tolerated replacements at conserved positions, including the single absolutely (F109) and 10 partially (F26, N35, S45, Y49, S56, Y64, Y70, F82, C84 and F107) conserved residues (Fig. 3). We also tested replacements of three poorly conserved residues (S25, S30 and Y32) that served as controls. Furthermore, we also abolished nsp12 expression by replacing its codons 6–8 with three consecutive translation termination codons (STOP mutant). The engineered cDNA clones were used for *in vitro* transcription, yielding full-length RNA that was subsequently electroporated into BHK-21 cells. The effects of the replacements were first assessed on the level of viral protein expression by immunofluorescence microscopy utilizing antibodies against nsp3 and the structural nucleocapsid (N) protein. Furthermore, we monitored the production of virus progeny by harvesting transfected cell culture supernatants and performing plaque assays (Table 1).

**Table 1.** EAV nsp12 mutants and their phenotypes Representative results of two independent datasets obtained by two different researchers.

					Immunofluorescence assay				Titre (p.f.u. ml^− 1^)		
Group^*^	Mutant	Observed amino acid variation†	WT sequence	Mutated sequence	14 h p.t.	24 h p.t.	48 h p.t.	68 h p.t.	14 h p.t.	48 h p.t.	nsp12 sequence of P1 virus‡
	WT				+	+	+	+	3 × 10^6^	2 × 10^8^	nd
4	S25A	FYTSARC	UCA	GCU	+	+	+	+	3 × 10^6^	2 × 10^7^	Mutation retained
2	F26A	FILV	UUC	GCA	–	–	–	–	< 20	< 20	nd
	F26Y	FILV	UUC	UAU	–	–	+	+	< 20	4 × 10^7^	Reversion
4	S30A	SLVIMD	UCA	GCU	+	+	+	+	6 × 10^5^	6 × 10^7^	Mutation retained
4	Y32A	IMAFRCY	UAC	GCA	–	+	+	+	20	5 × 10^5^	Y32V
	Y32F	IMAFRCY	UAC	UUU	+	+	+	+	5 × 10^8^	2 × 10^8^	Mutation retained
2	N35A	NS	AAC	GCU	–	–	–	+	< 20	< 20	Reversion
	N35D	NS	AAC	GAU	+	+	+	+	1 × 10^3^	1 × 10^8^	Reversion
4	S45A	SAG	UCA	GCU	+	+	+	+	2 × 10^7^	5 × 10^7^	Mutation retained
	S45T	SAG	UCA	ACC	+	+	+	+	1 × 10^3^	1 × 10^8^	Reversion
3	Y49A	YFW	UAC	GCA	–	–	–	–	< 20	< 20	nd
	Y49F	YFW	UAC	UUU	+	+	+	+	6 × 10^5^	4 × 10^7^	Mutation retained
4	S56A	ST	UCA	GCU	+	+	+	+	2 × 10^7^	4 × 10^7^	Mutation retained
	S56T	ST	UCA	ACC	+	+	+	+	4 × 10^7^	2 × 10^7^	Mutation retained
3	Y64A	YFWK	UAU	GCA	–	–	–	–	< 20	< 20	nd
	Y64F	YFWK	UAU	UUC	+	+	+	+	6 × 10^7^	2 × 10^8^	Mutation retained
3	Y70A	YFV	UAU	GCA	–	–	–	–	< 20	< 20	nd
	Y70F	YFV	UAU	UUC	+	+	+	+	4 × 10^7^	4 × 10^7^	Mutation retained
3	F82A	FYI	UUC	GCA	–	–	–	–	< 20	< 20	nd
	F82Y	FYI	UUC	UAU	+	+	+	+	1 × 10^8^	1 × 10^8^	Mutation retained
2	C84Y	CYAF	UGC	UAU	+	+	+	+	3 × 10^2^	3 × 10^7^	Reversion
1	F107A	FYC	UUC	GCA	–	–	–	–	< 20	< 20	nd
	F107Y	FYC	UUC	UAU	–	+§	+§	–	< 20	< 20	nd
1	F109A	F	UUC	GCA	–	–	–	–	< 20	< 20	nd
	F109Y	F	UUC	UAU	–	–	–	–	< 20	< 20	nd
	STOP		UCA	UGA	–	–	–	–	< 20	< 20	nd
			GCA	UGA							
			CUA	UGA							

* Groups are according to residues rather than mutants, as defined in the text.

† See Fig. 3.

‡ P1 virus was generated by infection of fresh BHK-21 cells with supernatant harvested at 68 h p.t. (for stable mutants) or the earliest positive time point in immunofluorescence microscopy (for reverting mutants and Y32V). ND, Not determined.

§ Non-spreading.

Neither protein expression nor progeny production was observed for the STOP mutant, indicating that nsp12 performed an indispensable function during virus replication. Alternatively, the truncation of nps12 may have affected virus viability indirectly, e.g. by impairing proteolytic cleavage of the nsp11/nsp12 junction, which might be detrimental to the activity of the nsp11 endoribonuclease. This concern was addressed by replacing individual nsp12 residues.

The 14 residues probed by making 25 mutants could be classified into four groups based on the impact of their replacement. The first group included residues F107 and F109, with the four mutants carrying alanine or (more conservative) tyrosine substitutions at these positions not producing any virus progeny. Interestingly, in contrast to both alanine mutants and F109Y, which also did not produce viral proteins, immunofluorescence signal for nsp3 and N protein was detected for F107Y at 24 and 48 h post-transfection (p.t.), with a stronger signal being observed at the earlier time point. Collectively, these results showed that F107 or F109 were most strongly constrained in EAV and indicated a vital role of these residues in virus viability.

The second group comprised residues F26, N35 and C84, which appeared to be only slightly less important than the aforementioned F107 and F109, based on the phenotype of five mutants. Alanine substitutions at position F26 and N35 were either lethal (F26A) or severely detrimental (N35A), whereas tyrosine or aspartate substitutions of these residues (F26Y and N35D) were compatible with at least some residual replication, which allowed early reversion of these mutants. Similarly, the C84Y mutant also reverted, which was notable given the presence of a tyrosine at this position in most other arteriviruses.

**Fig. 6.**
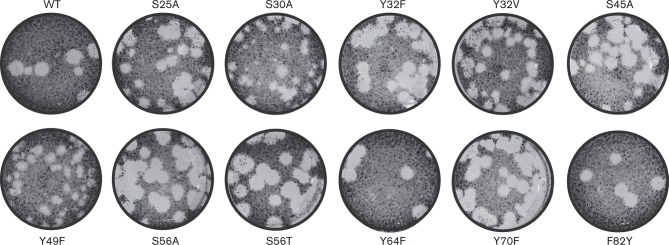
Plaque phenotypes of viable EAV nsp12 mutants. Virus-containing supernatants obtained 48 h p.t. were serially diluted and used to infect BHK-21 cells. After 72 h the cells were fixed with 4 % formaldehyde and stained with crystal violet.

In contrast to the above results, EAV tolerated replacements by another aromatic residue at four other partially conserved aromatic residues, Y49, Y64, Y70 and F82, which form group 3. These virus mutants were stable and yielded progeny titres up to 1 log below that of the WT control. Interestingly, although the titre of Y49F was not very different from that of the parental virus, this mutant exhibited a small-plaque phenotype (Fig. 6). In contrast, alanine substitutions at these positions were again lethal.

The replacement – more or less conservative – of all residues mentioned thus far had a moderate to severe impact on virus replication. In contrast, the fourth group included five residues whose replacement neither affected viral protein production nor progeny titres. As expected, this group included the three poorly conserved control residues (S25, S30 and Y32). Nevertheless, S30A exhibited a small-plaque phenotype (Fig. 6). Unexpectedly, we also repeatedly observed the pseudo-reversion of Y32A to Y32V, which required only a single nucleotide change. Although valine is not amongst the naturally occurring amino acid residues at this position (Fig. 3), a hydrophobic residue is observed in several arteriviruses other than EAV. In addition to substitutions of these control residues, EAV also tolerated the substitution of S56 with alanine or threonine. Given the strict conservation of serine and threonine, this lack of impact was the expected outcome for S56T, but was rather surprising for S56A. Finally, S45A was stable and indistinguishable from the parental virus, whilst S45T reverted. Together with the sequence variation at this position, which is limited to the small amino acids glycine, alanine and serine, this probably indicates a certain degree of steric hindrance by any residue larger than serine. Overall, the observed mutant phenotypes were compatible with the natural variation observed at the respective positions, with the possible exception of the C84Y mutant. These correlations supported the multiple sequence alignment (MSA) of the highly variable nsp12 and suggested that EAV replication in BHK-21 is a faithful model system for probing nsp12 function by mutagenesis.

Both mutants displaying a small-plaque phenotype (S30A and Y49F), as well as the unexpected Y32V pseudo-revertant, were further investigated in terms of growth kinetics and accumulation of intracellular viral RNA (not shown). Compared with the WT control, S30A and Y49F demonstrated a slight delay in replication early during infection [8 h post-infection (p.i.)], but eventually reached comparable titres by 24 h p.i. In line with this finding, the amounts of genomic and subgenomic mRNA at 8 h p.i. were reduced for both mutants. Whether this was due to a decreased synthesis or lower stability of their RNAs remains to be investigated. In contrast, the stable Y32V mutant was essentially indistinguishable from the WT control both in terms of growth kinetics and amounts of RNA produced.

## Discussion

The most conserved ORF1b of nidoviruses encodes only two proteins that have not been studied before in any virus. Our study aimed to address this knowledge gap for one of these proteins, arterivirus nsp12. It established (i) the exceptional divergence of nsp12, (ii) the lack of strong bioinformatics and biochemical support for nsp12 being an MTase, and (iii) the fact that nsp12 is essential for arterivirus replication.

So far, none of the four enzymic activities required for conventional cap-1 synthesis, or any of the known alternative capping strategies, has been uncovered for arteriviruses, although arteriviral mRNAs are presumed to be capped. In the conserved relative arrangement of replicative enzymes within nidovirus pp1a and pp1ab, the unique arterivirus nsp12 is encoded in a genome position equivalent to that of the coronavirus 2′-*O*-MTase, which is also conserved in invertebrate nidoviruses (Fig. 1). We thus asked whether this so far uncharacterized subunit may represent an MTase, potentially able to perform both methylation reactions as, for example, the flavivirus NS5 MTase domain is (Zhou *et al.*, 2007). Upon our bioinformatics analysis of nsp12 sequences, we found that this subunit, similar to the N7-MTase residing in coronavirus nsp14, is enriched with (partially) conserved aromatic amino acids and is predicted to fold in alternating α-helices and β-strands (Fig. 3). Nevertheless, no statistically significant similarity was found between nsp12 and other MTases of viral or cellular origin.

When we subsequently sought to verify our hypothesis using an *in vitro* MTase assay, we could not detect activity above background for recombinant EAV nsp12, whereas our positive controls confirmed the functionality of the assay. To explain this lack of activity, we argued that, as for coronavirus nsp16, a second EAV protein may be required to form a functional MTase complex. By analogy with the coronavirus nsp10 co-factor, we tested the possibility that this second protein might be encoded just upstream of the ORF1a/1b ribosomal frameshift site. We thus expressed and purified nsp7α and nsp7β, as well as three polyprotein cleavage intermediates containing these two proteins, and included them in our assays (Fig. 5). However, in these extended assays we also could not detect any MTase activity. This could be for multiple reasons. (i) The proteins tested here may not be the correct co-factors or may be unable to properly associate with nsp12 under the conditions employed. (ii) More than one co-factor may be needed to spur nsp12's MTase activity or different RNA substrates containing specific sequences may be required. (iii) It may be that nsp12, which is smaller than other viral MTases, does not possess MTase activity, in which case other hypotheses about its function should be considered (see below).

To explore nsp12's relevance for arterivirus replication, we engineered one truncation and 25 point mutations of EAV nsp12, and launched the corresponding mutant genomes in BHK-21 cells. Reflecting the conservation of several aromatic residues in arteriviruses, substitution with alanine was not tolerated in any of the cases, whereas more conservative substitutions maintaining the residue's aromatic nature were tolerated in most of the partially conserved positions (Table 1). The only exception was F107Y, which interestingly showed a certain level of protein expression, but did not produce infectious progeny. As two arteriviruses distantly related to EAV, LDV and PRRSV genotype 1, naturally encode a tyrosine at this position (Fig. 3), this result suggests an epistatic interaction between residue 107 and other unknown residue(s). EAV also did not tolerate a block of nsp12 expression (STOP mutant) or the replacement of its single absolutely conserved nsp12 residue, F109, with alanine or tyrosine. This phenotype could be explained by a *trans*-dominant negative effect of these mutations on an interaction partner of nsp12, if this partner is essential for EAV replication. This explanation is also compatible with the non-viable phenotype of several other mutants and suggests a particularly important role of the most constrained and proximal F107 and F109 in such a putative interaction.

The fact that EAV does not tolerate substitution of its single invariant nsp12 residue stands in remarkable contrast to phenotypes described for mutants of the invariant residues of the NendoU or 2′-*O*-MTase of nidoviruses (Kang *et al.*, 2007; Menachery *et al.*, 2014; Posthuma *et al.*, 2006; Züst *et al.*, 2011), which are both more strongly conserved than nsp12. In these studies, alanine substitutions of absolutely conserved putative active-site residues resulted in lower virus progeny titres and in part in small-plaque phenotypes in cell culture, but did not entirely abolish virus replication.

In conclusion, our combined results may be most compatible with the notion that nsp12 is not an MTase and possibly not even an enzyme, but rather a co-factor of an essential component of the arterivirus replicase. In this context, a future in-depth analysis of the nsp12 interaction network could be most informative. If nsp12 is not an MTase, this activity must be provided by another protein, but it is unlikely to be one of the three other ORF1b proteins, which are known to possess non-MTase enzymatic activities. This implies that arteriviruses may be (very) different from other nidoviruses with respect to either the nature of the 5′ end of their mRNAs and/or the mechanism generating it. We note that the presence of a 5′-terminal cap-1 structure was reported for the SHFV genome (Sagripanti *et al.*, 1986), but that monophosphates were claimed to present at the 5′ end of LDV mRNAs (Chen *et al.*, 1994), calling for additional studies to resolve the apparent conflict. Finally, the possibility of cap-snatching, the strategy employed by some families of negative-stranded RNA viruses (Fujimura & Esteban, 2011; Mir *et al.*, 2008; Reich *et al.*, 2014), may be explored for arteriviruses. This mechanism might accommodate the nsp11 NendoU as endoribonuclease and nsp12 as a cap-binding protein, which would connect coronavirus nsp16 and arterivirus nsp12 to a common target in an unorthodox way.

## Methods

### Bioinformatics

Genomes of members of the families *Arteriviridae* and *Coronaviridae* were retrieved from GenBank (Benson *et al.*, 2013) and RefSeq (Pruitt *et al.*, 2014) using the haygens (homology-annotation hybrid retrieval of genetic sequences) tool (http://veb.lumc.nl/HAYGENS). Codon-based MSAs of virus genomes were produced using the Viralis platform (Gorbalenya *et al.*, 2010), and assisted by hmmer 3.1 (Finn *et al.*, 2011), Muscle 3.8.31 (Edgar, 2004) and clustal w 2.012 (Larkin *et al.*, 2007). Only one virus per established or tentative species, which were defined with the help of DEmARC1.3 (Lauber & Gorbalenya, 2012), was retained for bioinformatics analyses. snad (Sidorov *et al.*, 2009) was used to retrieve information about genomes. To reveal the full extent of similarity between pairs of alignments, they were converted into HMM profiles, which were compared and visualized in a dot-plot fashion using a routine in HH-suite 2.0.15 (Remmert *et al.*, 2012; Söding, 2005). Distribution of similarity density in alignments was plotted using r package Bio3D (Grant *et al.*, 2006) under the conservation assessment method similarity, substitution matrix Blosum62 (Henikoff & Henikoff, 1992) and a sliding window of 11 alignment columns. HH-suite 2.0.15 (Remmert *et al.*, 2012; Söding, 2005) was used to search for homologues amongst profiles in the Pfam-A database (Finn *et al.*, 2014); the secondary structure of proteins was predicted by applying JPred 3 (Cole *et al.*, 2008) and psipred (Buchan *et al.*, 2013) to MSAs. The MSAs were converted into figures using ESPript (Robert & Gouet, 2014). Reconstruction of phylogenetic trees was performed using PhyML 3.0, with the WAG amino acid substitution matrix (Whelan & Goldman, 2001), allowing substitution rate heterogeneity amongst sites (four categories) and 1000 iterations of non-parametric bootstrapping (Guindon *et al.*, 2010). PPDs between viruses were calculated from protein trees using r package ape (Paradis *et al.*, 2004). Linear regression was calculated using r package stats (R Development Core Team, 2011).

### Reverse genetics of EAV

Mutations specifying alanine and conservative replacements of (partially) conserved and control residues in nsp12 were generated using the QuikChange protocol. In all cases translationally silent marker mutations were introduced to allow discrimination between (partial) reversion of mutants after transfection and (possible) contamination with WT virus. Mutated gene fragments were introduced into full-length cDNA clone pEAV211 (van den Born *et al.*, 2004), a pEAV030 derivative (van Dinten *et al.*, 1997), using appropriate shuttle vectors and restriction enzymes. The presence of the mutations was confirmed by sequencing. pEAV211 DNA was *in vitro* transcribed and RNA was purified by LiCl precipitation. RNA was transfected into BHK-21 cells as described previously (Nedialkova *et al.*, 2010). Transfected cells were monitored by immunofluorescence microscopy until 68 h p.t. using antibodies directed against EAV nsp3 and N protein as described previously (van der Meer *et al.*, 1998). To monitor the production of viral progeny, plaque assays were performed with supernatants collected at 14 and 48 h p.t. or during the first 24 h p.i. to determine growth kinetics, as described previously (Nedialkova *et al.*, 2010). To verify the presence of the introduced mutations or reversions in viable mutants, fresh BHK-21 cells were infected with supernatants harvested at time points at which transfected cells were positive in immunofluorescence microscopy. RNA was isolated after 18 h or when a cytopathic effect was detected. Finally, the nsp12-coding region was amplified by reverse transcription (RT)-PCR using random hexameric primers in the RT step and EAV-specific primers for the PCR. PCR fragments were purified and sequenced.

### Protein expression and purification

N- and C-terminal His-tag fusion proteins of WT nsp12 were expressed from a pDEST vector. Plasmids were transformed into *E. coli* BL21(DE3) and cells were grown in Luria Broth (LB) with 100 μg ampicillin ml^− 1^ at 37 °C until OD600 0.7. Expression was induced after addition of 0.5 mM IPTG and cells were grown for further 4 h at 37 °C.

EAV ORF1a-encoded proteins were expressed with N-terminal ubiquitin and C-terminal His-tags from pASK vectors (Gohara *et al.*, 1999). Plasmids were transformed into *E. coli* C2523 containing the pCG1 plasmid, which leads to constitutive expression of the ubiquitin-specific protease UBP1. Cells were grown in LB with 100 μg ampicillin ml^− 1^ and 34 μg chloramphenicol ml^− 1^ at 37 °C until OD600 0.7. Expression was induced after addition of 200 ng anhydrotetracycline ml^− 1^ and cells were grown for another 18 h at 20 °C. All pellets were harvested by centrifugation and stored at − 20 °C until further use.

Proteins were batch purified by metal affinity chromatography using Co^2+^ (Talon beads). All steps were performed at 4 °C or on ice. Cells were resuspended in nsp12 resuspension buffer (20 mM HEPES, pH 7.5, 5 mM β-mercaptoethanol) or co-factor resuspension buffer [20 mM HEPES, pH 7.5, 10 % glycerol (v/v), 5 mM β-mercaptoethanol] containing 500 mM NaCl and Roche complete EDTA-free protease inhibitor cocktail. Lysis was achieved by 30 min incubation with lysozyme (0.1 mg ml^− 1^). Genomic DNA was sheared during four sonication cycles of 10 s with intermittent cooling. Cell debris was removed by centrifugation at 20 000 ***g*** for 20 min. Cleared supernatants were incubated with an appropriate amount of Talon beads for 1 h under slow rolling. Beads were collected and washed four times for 15 min with a 20-fold volume of the respective resuspension buffer supplemented with 10 mM imidazole, and first 500 mM, then 250 mM and finally twice 100 mM NaCl. Proteins were eluted with the respective resuspension buffer containing 300 mM imidazole and 100 mM NaCl. Elution fractions were examined by SDS-PAGE, pooled and dialysed against 20 mM HEPES, pH 7.5, 100 mM NaCl, 25 % glycerol, 1 mM DTT. All proteins were stored at − 20 °C. Typical yields were 1–2 mg l^− 1^ culture for all proteins. Protein concentrations were calculated based on theoretical extinction coefficients and *A*_280_.

Gel filtration of nsp12 was performed on a Superdex 75 10/300 GL gel filtration column with 10 mM sodium phosphate buffer, pH 6.0, 100 mM NaCl, 1 mM DTT at 4 °C and a flow rate of 0.5 ml min^− 1^.

### MTase assay

MTase assays were performed essentially as described previously (Bouvet *et al.*, 2010). Proteins at the indicated final concentrations were incubated at 30 °C for 30, 60 or 180 min in a buffer containing 20 mM HEPES, pH 7.5, 5 mM DTT, 0.5 mM MgCl_2_, 0.5 mM MnCl_2_, 10 μM *S*-adenosylmethionine, 2 μM capping substrate and 1 × 10^3^ Bq *S*-[methyl-^3^H]adenosylmethionine μl^− 1^. Additionally 7.5 mM NaCl was carried over from the protein storage buffer. Vaccinia virus capping enzyme (New England Biolabs) was incubated in the buffer supplied by the vendor. A 10-fold volume of ice-cold *S*-adenosylhomocysteine (100 μM) was added to stop the reaction. Samples were spotted on DEAE filtermats (Perkin Elmer), which were subsequently washed twice with 10 mM ammonium formate, pH 8.0, then twice with water and finally with ethanol. Filtermats were cut and radioactivity was measured by scintillation counting.

## Supplementary Data

273 Supplementary Data
