# Supplementary material for: Arterivirus nsp12 versus the coronavirus nsp16 2′-O-methyltransferase: comparison of the C-terminal cleavage products of two nidovirus pp1ab polyproteins
Source: J Gen Virol. 2015 Sep;96(Pt 9):2643–55. doi: 10.1099/vir.0.000209 (PMC7081073; doi:10.1099/vir.0.000209)
Supplement: Supplementary file 1 — Supplementary Data [file jgv-96-2643-s001.pdf]

Supplementary Material

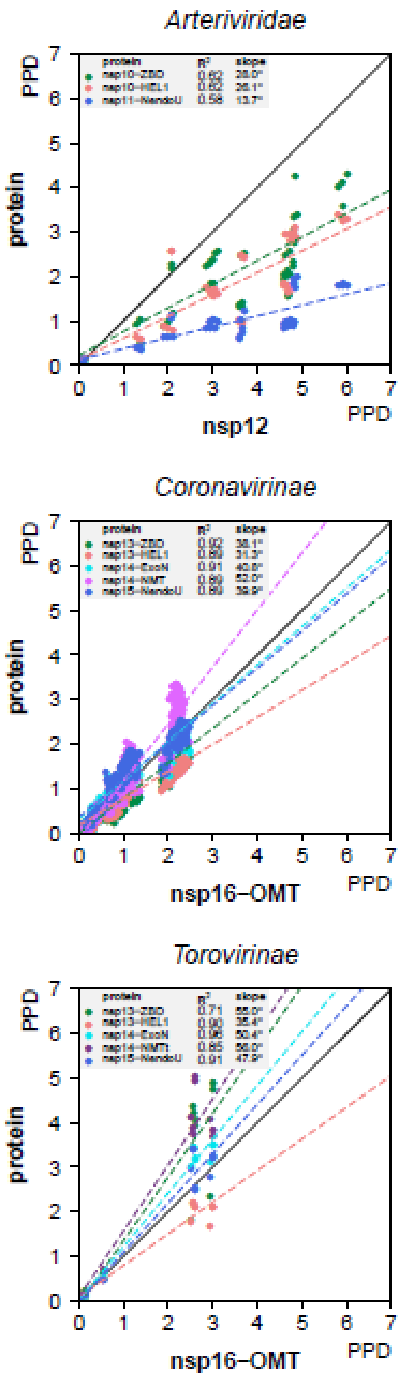

Supplementary Figure 1. Relative scale of divergence of nsp12 of the *Arteriviridae* and (putative) nsp16 of the *Coronavirinae* and *Torovirinae*. Shown are three (sub)family-specific two-dimensional scatter plots that compare PPDs of the most C-terminal protein (nsp12 or nsp16-OMT, x-axis) versus PPDs of other proteins/domains of ORF1b starting from the ZBD (detailed in inset, y-axis). PPDs were calculated from PhyML trees for separate proteins. Dashed lines, linear regressions fit in respective (color matching) dot distributions with R^2 and slope values being detailed in the inset panels.

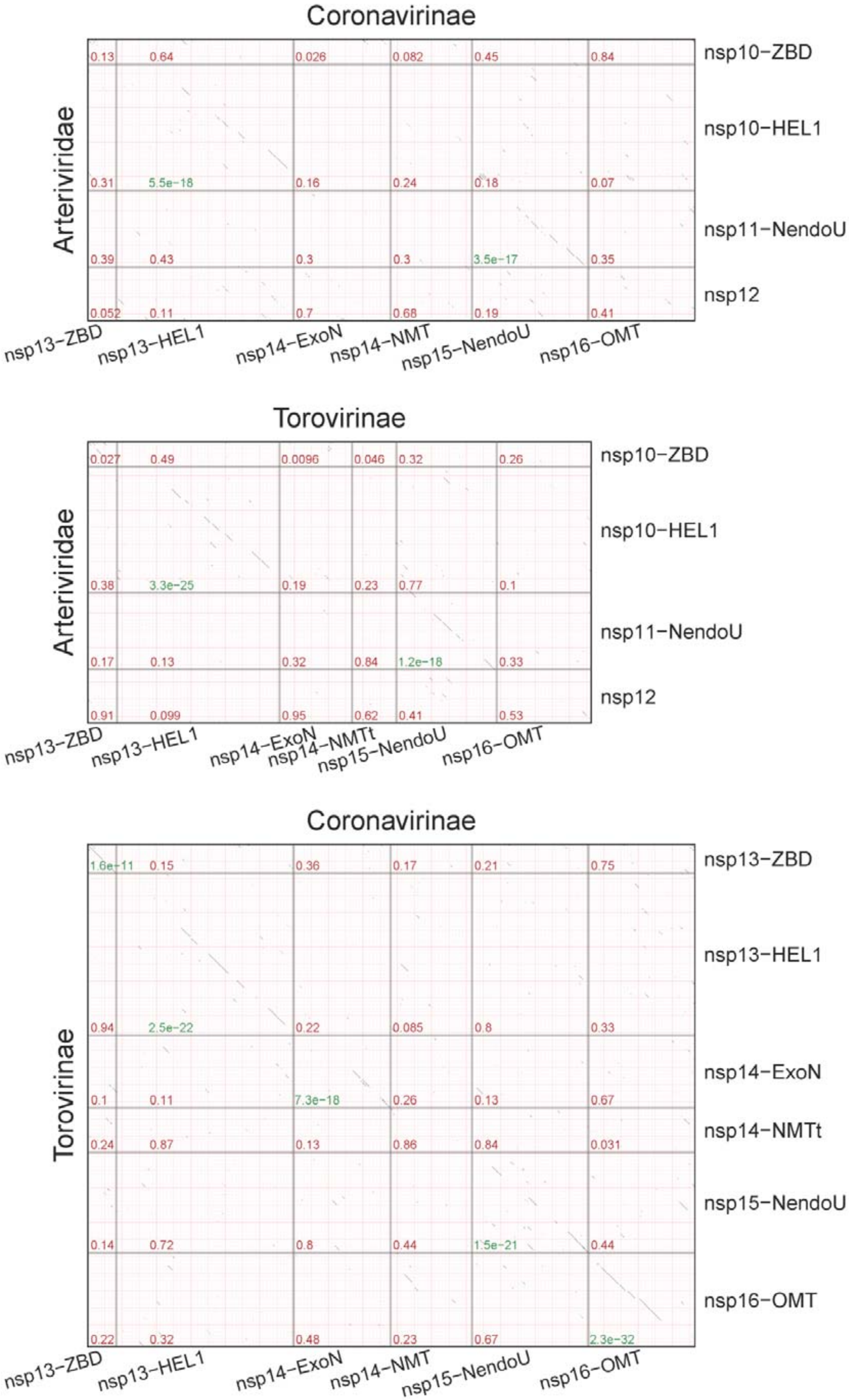
Coronavirinae
Arteriviridae
nsp10−ZBD
nsp10−HEL1
nsp11−NendoU
nsp12
nsp13−ZBD
nsp13−HEL1
nsp14−ExoN
nsp14−NMT
nsp15−NendoU
nsp16−OMT
0.13 0.64 0.026 0.082 0.45 0.84
0.31 5.5e−18 0.16 0.24 0.18 0.07
0.39 0.43 0.3 0.3 3.5e−17 0.35
0.052 0.11 0.7 0.68 0.19 0.41
Torovirinae
Arteriviridae
nsp10−ZBD
nsp10−HEL1
nsp11−NendoU
nsp12
nsp13−ZBD
nsp13−HEL1
nsp14−ExoN
nsp14−NMTt
nsp15−NendoU
nsp16−OMT
0.027 0.49 0.0096 0.046 0.32 0.26
0.38 3.3e−25 0.19 0.23 0.77 0.1
0.17 0.13 0.32 0.84 1.2e−18 0.33
0.91 0.099 0.95 0.62 0.41 0.53
Coronavirinae
Torovirinae
nsp13−ZBD
nsp13−HEL1
nsp14−ExoN
nsp14−NMTt
nsp15−NendoU
nsp16−OMT
nsp13−ZBD
nsp13−HEL1
nsp14−ExoN
nsp14−NMT
nsp15−NendoU
nsp16−OMT
1.6e−11 0.15 0.36 0.17 0.21 0.75
0.94 2.5e−22 0.22 0.085 0.8 0.33
0.1 0.11 7.3e−18 0.26 0.13 0.67
0.24 0.87 0.13 0.86 0.84 0.031
0.14 0.72 0.8 0.44 1.5e−21 0.44
0.22 0.32 0.48 0.23 0.67 2.3e−32

Supplementary Figure 2. Analysis of co-conservation of C-terminal parts of ORF1b of the *Arteriviridae*, *Coronavirinae,* and *Torovirinae*. Shown are three pair-wise MSA-based HMM-HMM plots comparing parts of ORF1b starting from the ZBD of three origins. The position of proteins and some domains are indicated. Each MSA was converted to an HMM profile, three possible pairs of obtained HMMs were aligned with the help of HH-suite 2.0.15 software (Remmert *et al.*, 2012;Soding, 2005). The presence of similarity above the threshold of 0.3 is recorded with a dot. Diagonal persistence of dots is strong evidence for statistically significant similarity (homology) of a protein pair. E value of similarity is indicated for each protein pair compared. E values below 0.001 are indicated in green, those above in red.
